# Collecting duct carcinoma with retroperitoneal mass as initial presentation: a rare case report

**DOI:** 10.1186/s12894-023-01295-6

**Published:** 2023-07-26

**Authors:** Rupei Ye, Yehui Liao, Tian Xia, Xinfeng Zhang, Qiyi Lu, Xiuli Xiao

**Affiliations:** 1 Department of Pathology, The Affiliated Hospital of Southwest Medical University, Luzhou, Sichuan province China; 2 Department of Orthopaedics, The Affiliated Hospital of Southwest Medical University, Luzhou, China; 3 Department of Pathology, Yanyuan County People’s Hospital, XiChang, China

**Keywords:** Collecting duct carcinoma, Lymph node metastasis, Case report

## Abstract

**Background:**

Collecting duct carcinoma (CDC) is a rare renal tumor, originating from the distal collecting duct. CDC rarely presents as a primary tumor outside the renal system.

**Case presentation:**

In this study, we report a rare case of collecting duct carcinoma, with an initial presentation of retroperitoneal lymph node metastasis, and no identifiable primary renal tumor on CT, at the time of diagnosis. The patient was a 64-year-old man presenting with lower back pain. Preoperative CT showed a round, soft tissue mass, measuring 6.7 × 4.4 × 3.3 cm, in the left retroperitoneum with no exact occupying lesion in the left kidney. Clinically, ectopic pheochromocytoma was considered to be a differential diagnosis, and tumor resection was performed. Postoperative pathological results demonstrated that the mass was a fused lymph node, and the tumor cells were destroying the structure. The final diagnosis was lymph node metastatic collecting duct carcinoma, by histology and immunohistochemistry. No further treatment was performed as no space occupying lesion was found in the kidney. Three months later, CT was reexamined, and a mass of 3.6 cm in diameter, was found in the lower left kidney, along with multiple soft tissue masses, in the left renal hilum. Considering recurrence or metastasis, the patient was recommended to undergo surgical treatment, but the patient refused. Four months later, CT was re-examined. The tumor had rapidly progressed but the patient refused treatment again. As per the author’s press release (eleven months after the first discovery), the patient is still alive.

**Conclusion:**

CDC is a rare malignant renal carcinoma, with a high chance of rapid progress, regional lymph nodes involvement and metastasis. It presents diagnostic challenges to clinicians and pathologists, particularly, in the absence of radiographically detectable intrarenal lesions. Definite diagnosis is based on pathological examination combined with immunohistochemical staining.

## Background

Collecting duct carcinoma is a rare type of renal cell carcinoma, accounting for less than 2% of all cases of renal cell carcinoma [1, 2]. Due to the rapid progression of the disease and extensive metastasis to surrounding lymph nodes, most patients have an extremely low prognosis, with a median survival of less than 2 years. Early diagnosis seems to be the only factor for prolonged survival [3]. Although the disease has immunohistochemical features, its rarity makes it difficult to be identified histologically, and it also requires differentiation from other renal cell and urothelial carcinomas. Literature has reported that collecting duct carcinoma is usually located near the kidney or renal pelvis. However, with lymph node metastasis as the initial diagnosis, no renal occupying lesion was found at presentation and, has not been described either.

## Case presentation

### Clinical information

**Fig. 1:**
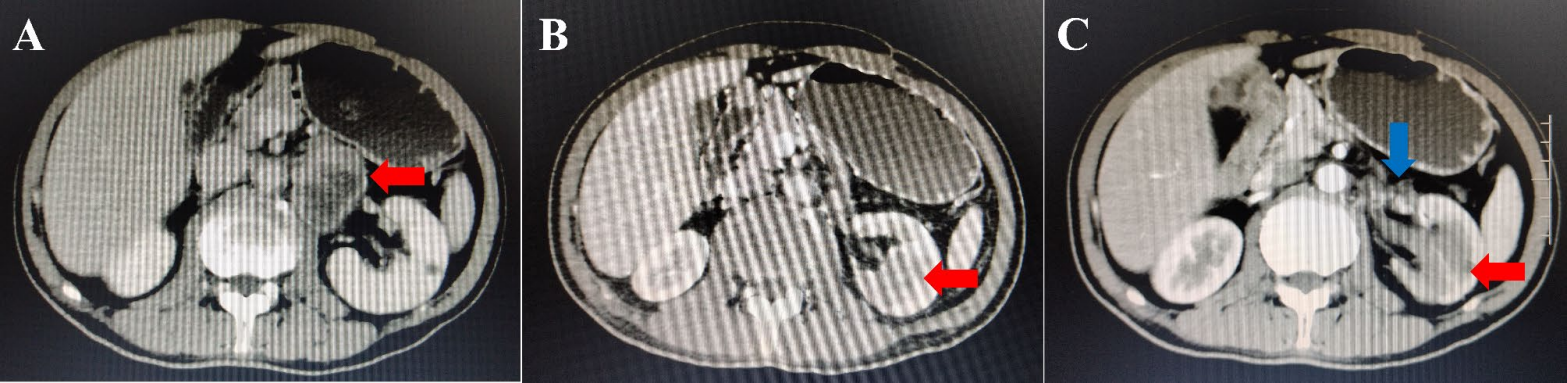
**A.** Contrast-enhanced CT shows a tuberculous retroperitoneal mass (red arrow) on the left side of the kidney. **B.** 3 months later, at the first abdominal CT review, a mass about 2.5 cm in size was found occupying the left hilum (red arrow). **C.** 5 months later, the second abdominal CT review revealed a large and small 3.6 cm soft tissue mass under the left kidney (red arrow) and multiple soft tissue shadows in the renal portal area (blue arrow)

A 64-year-old male presented with left upper quadrant pain of 1 week duration. The pain was worse when standing and relieved on lying down. On physical examination, no palpable abdominal mass was found. Computed tomography (CT) scan of the abdomen revealed a 6.7 × 4.4 × 3.3 cm, heterogeneously enhancing circular mass in the left retroperitoneum (Fig. 1A). The enhanced scan showed uneven enhancement. The fat space around the mass was blurred and the left perirenal fascia was thickened. No lesion was seen in either of the kidneys. Moreover, there were no congenital renal abnormalities such as horseshoe kidney or ectopic kidney. The patient has a 2-year history of hypertension with the highest recorded blood pressure of 160/130mmHg and the patient reports taking regular medications. Based on clinical examination and radiological assessment, a clinical diagnosis of ectopic pheochromocytoma was made. Subsequently, tumor resection was performed through transperitoneal approach. After resecting the tumor, the resected specimens were immediately fixed in 10% formalin.

### Pathological examination

**Fig. 2:**
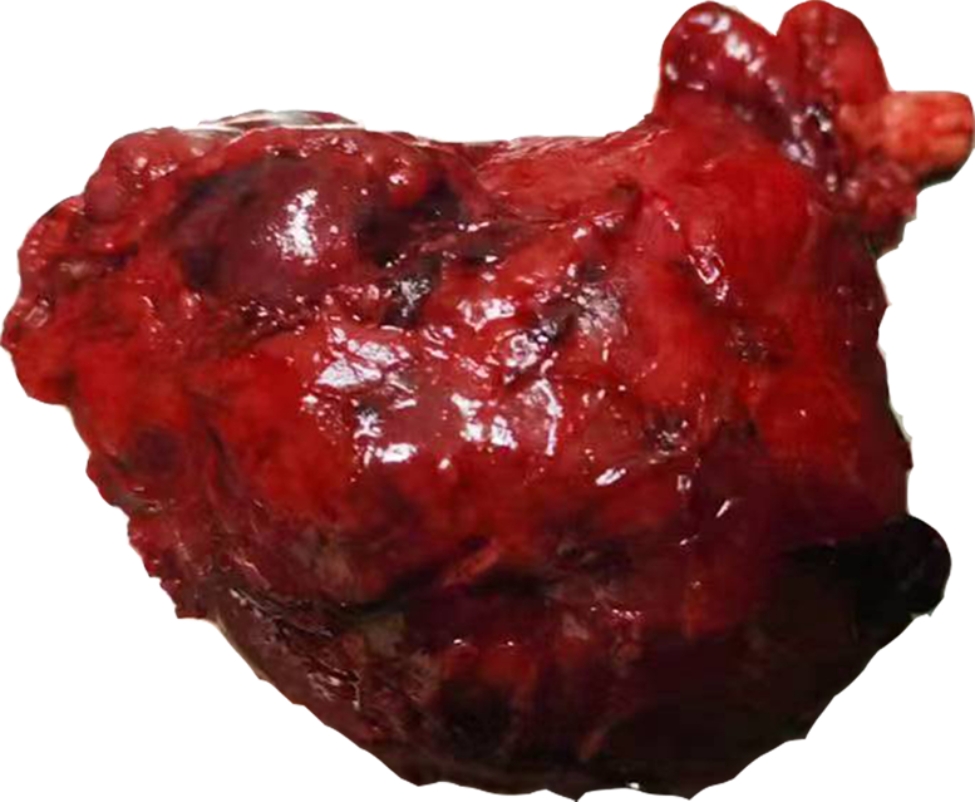
On gross examination of a well-delineated mass

**Fig. 3:**
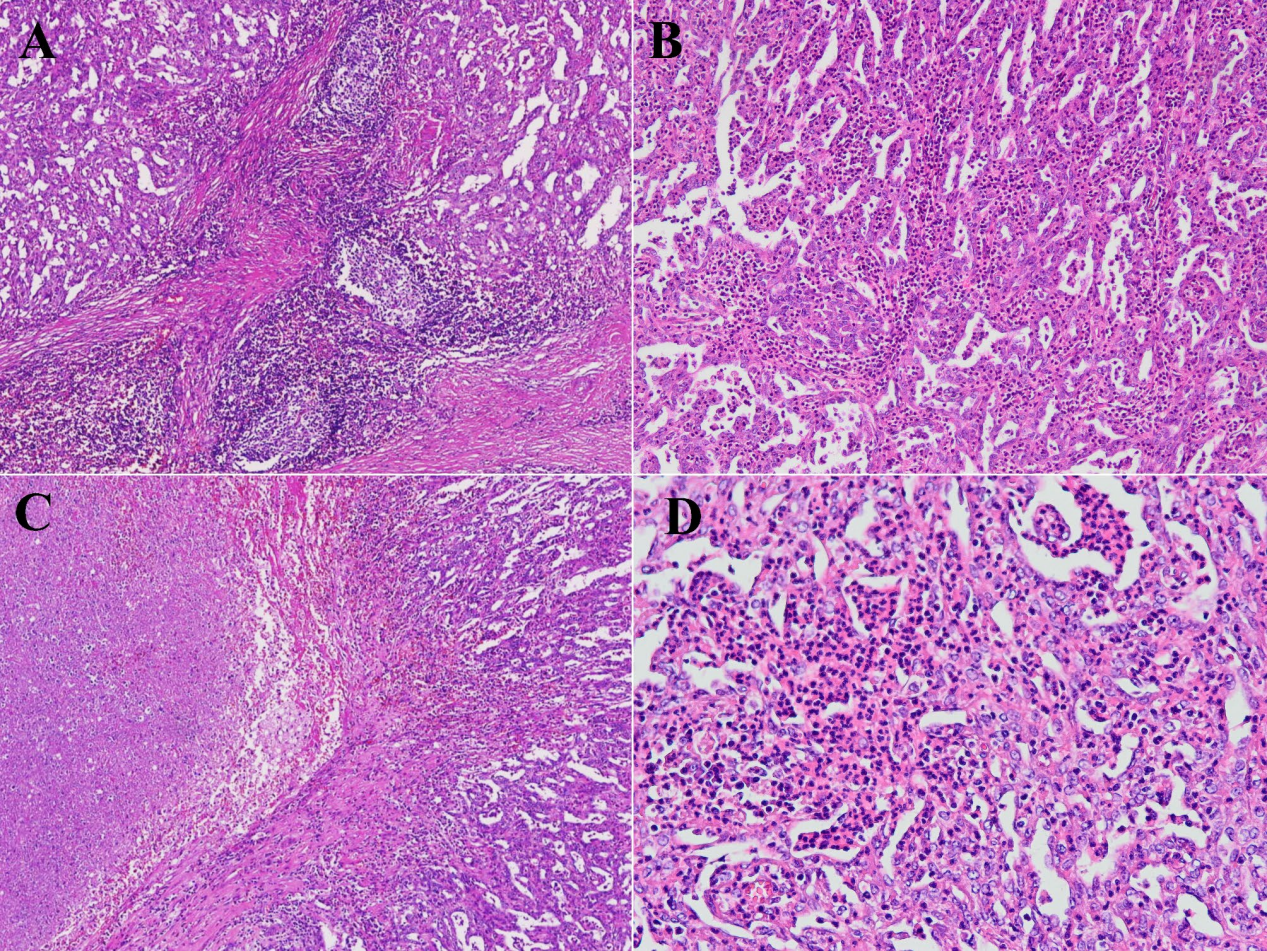
**A.** At low magnification, the tumor destroys the lymph node structure and a focal residual lymphatic follicle is seen. **B.** The tumors are arranged in tubular or tubular papillae, and there is a large amount of inflammatory cell infiltration in the interstitium. **C.** The tumor on the left shows extensive necrosis. **D.** At high magnification, the tumor cells were columnar or cubed, with unclear boundaries, abundant cytoplasm, deeply stained nuclei, and obvious nucleoli

**Fig. 4:**
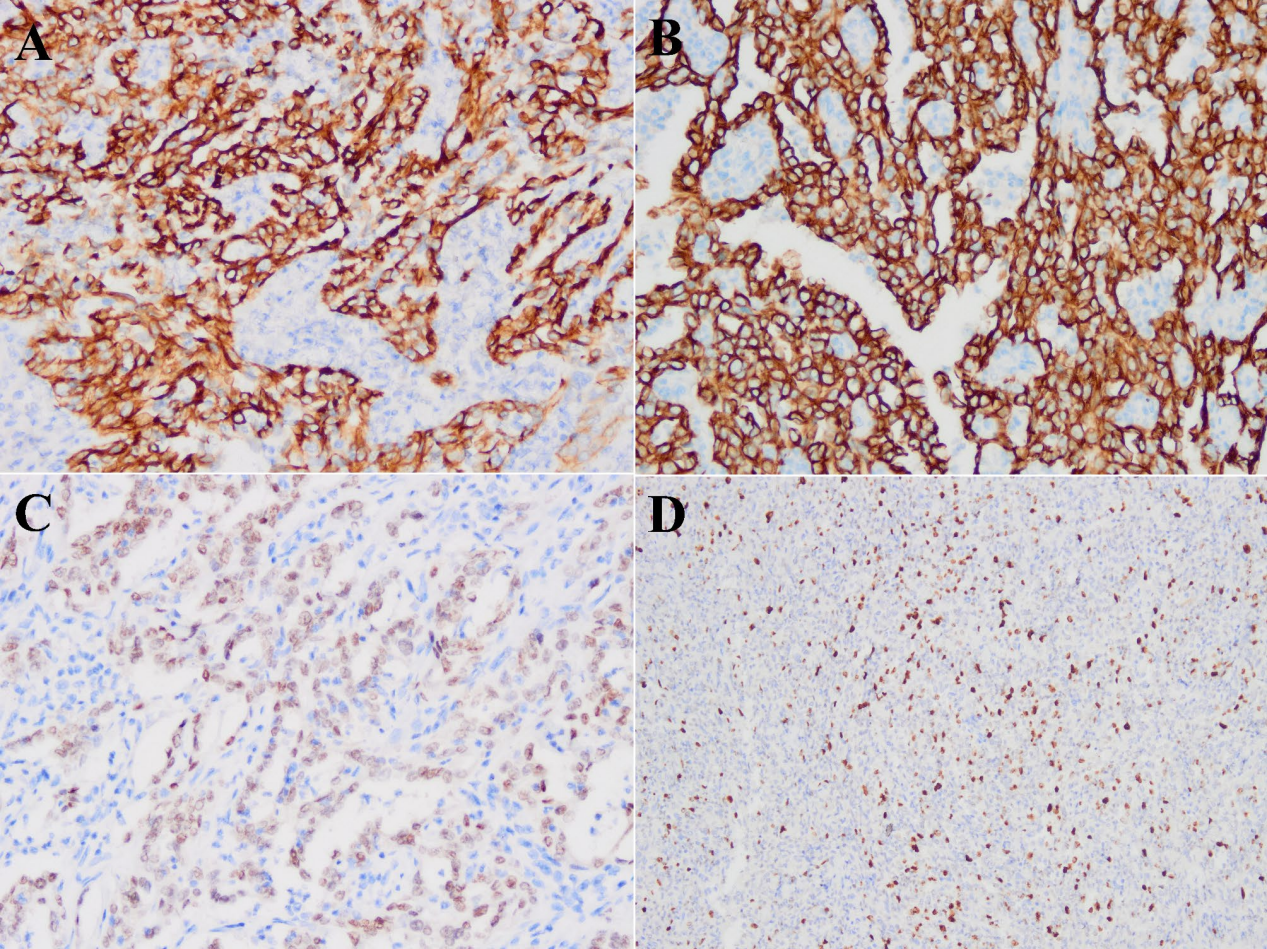
**A.** Tumor cells were positive for CK7. **B.** Tumor cells were positive for CK18. **C.** Tumor cells were positive for PAX8. **D.** The KI67 proliferation index of tumor cells was about 40%

On gross examination of a 10 cm well-delineated mass (Fig. 2), the section was grayish-white, solid, and hard. Microscopically, the mass had a partial envelope, with a few residual lymphatic follicles at the border (Fig. 3A). Most of the structure was destroyed by tumor cells. The tumor was mainly composed of hornlike tubules and tubular papillary structures, covered by a single layer of epithelium (Fig. 3B). Fibroconnective tissue hyperplasia is seen in the interstitium, with an infiltration of lymphocytes and neutrophils. The tumor is accompanied by mass necrosis (Fig. 3C). At higher magnification, tumor cells were found to be cuboid, columnar and shoe-shaped, with deeply stained nuclei, protrusion nucleoli, and pathological mitosis (Fig. 3D). No normal renal tissue was seen. The initial diagnosis was metastatic lymph node malignancy, and the differential diagnosis included renal collecting duct carcinoma, papillary renal cell carcinoma, medullary carcinoma, high-grade urothelial carcinoma, among others. An immunohistochemical study was performed, using the DaKo EnVision method. The tumor cells indicated a strong reactivity for CK7(Fig. 4A), CK19, CK18(Fig. 4B), PAX8(Fig. 4C), and EMA. To further exclude other tumor entities, we also stained for Melan-A, Inhibin-a, OCT3/4, Syn, CgA, which were all negative. The Ki-67 index focally reached to 30–40% of tumor cells (Fig. 4D). As no identifiable normal renal tissue was found, the possibility of this mass developing in a supernumerary kidney, was ruled out. Thus, combined with morphological and immunohistochemical evaluation, the final pathological diagnosis was metastatic collecting duct carcinoma of lymph nodes. No further nephrectomy was performed because the patient had no significant detectable masses, cysts or other abnormalities, in either kidney on enhanced CT. The patient was subsequently discharged and close follow-up was recommended.

### Following up

Three months later, the patient underwent abdominal CT examination in the outpatient department and was surprised to find a mass occupying shadow about 2.5 cm in size at the left renal hilum. Furthermore, multiple nodules of different sizes were observed retroperitoneally, with the largest lesion of 2.7 × 2.2 cm (Fig. 1B). The diagnosis was considered to be renal malignancies with retroperitoneal lymph node metastasis. The oncologist recommended radiation therapy and chemotherapy. At that time, the patient was extremely thin and lived in a rural area, making it difficult to travel back and forth. Therefore, the patient refused treatment. Five months later, the patient underwent another CT review in the outpatient department, which revealed a soft tissue mass with a diameter of 3.6 cm in the lower part of the left kidney and multiple soft tissue shadows in the renal portal area, which was considered to be renal malignant tumor with metastasis (Fig. 1C). Again, the patient refused treatment. Currently, the patient is still alive, 11 months after being diagnosed with collecting duct cancer.

## Discussion

Renal collecting duct carcinoma, considered to originate from the medullary collecting ducts of the kidney, is a very rare subtype of renal cell carcinoma, accounting for 0–3% of all renal malignancies [4]. The initial observation of the Bellini duct epithelial cells as the source of tumor development, was reported by Cromie W et al [5]. The 2016 WHO Classification of Tumors of the Urinary System, defined diagnostic criteria for this entity [6]. CDC is most often found in middle-aged and older patients and more often observed in men than in women [7]. More than half of the patients with collecting duct cancer, are known to have symptoms at the time of diagnosis. The most common symptom is gross hematuria, followed by back pain or palpable abdominal mass [8]. Moreover, tumors occurring on the left side have an advantage over those on the right side [4]. Unlike other renal cell carcinomas, CDC is highly aggressive with extensive metastasis to local lymph nodes. Upon diagnosis, up to 70% of patients have present with metastases to the lymph nodes, lungs and bone marrow, as the most common sites [7]. In this report, we report a collecting duct carcinoma, which originally presented a retroperitoneal mass on imaging. The renal lesions were undetected.

Through literature review, different hypotheses have been proposed to explain this pathogenesis in the absence of identifiable primary renal tumors.

Mansoor M [9] reported three cases of extra-renal, renal cell carcinoma and proposed that, the origin of renal tumors in the vicinity of the kidney may include three possible explanations:

1. The first is assumed to be derived from residual renal embryonic structures that are usually lost in adults, such as mesonephros, or possibly from ectopic renal tissue. For this reason, ectopic renal tissue is often seen in the tumor.
2. A second explanation is that the primary tumor in the kidney may have regressed. Johnson MT [10] reported a case of metastatic clear cell renal cell carcinoma located in the adrenal gland. There was a recognizable “degenerative region”, in renal imaging examination, before and after tumor resection, which was suspected to be primary “spontaneous regression” of the kidney.
3. A final explanation is that the primary tumor in the kidney is very small or cystic, without a solid part. Therefore, imaging modalities cannot detect very small primary renal lesions.
  This case falls into the third category, because, during follow-up, in a few months, the renal mass progressed rapidly.

Extrarenal manifestations of collecting duct carcinoma, are a challenge for clinicians and pathologists. Given the lack of specific imaging findings, distinguishing CDC from other tumors by imaging alone, is extremely challenging. Yu Z [11] described and analyzed the imaging features of 13 cases of CDC, and the results showed that the density of CDC in plain CT scans was higher than that of the surrounding normal tissue, fibroblast proliferation was obvious in the interstitial, the enhancement degree was lower than that of the renal parenchyma on the dynamic, enhanced scan, and the low signal on T2WI MRI was different from that of other RCCs. So it is important to be alert for CDC. Positron emission tomography-computed tomography (PET-CT) has certain limitations in the detection and diagnosis of kidney cancer, and for the CDC, it often exhibits high FDG uptake and is effective in the diagnosis of kidney tumor metastasis [11]. In view of this, PET-CT examination is recommended after radical nephrectomy to further clarify the systemic situation. The correct diagnosis still depends on the pathological examination. Neoplasms in the posterior peritoneum, adjacent to the kidney, including pheochromocytoma, medullary carcinoma, renal cell carcinoma, or poorly differentiated urothelial carcinoma, should be considered. The most common histomorphological structures observed in CDC, are acinus, tubular, tubular papillary and other growth patterns, with obvious fibrous connective tissue promoting response, inflammatory cell infiltration (mainly of lymphocytes and neutrophils in the interstitium), and the tumor is often accompanied by apoptosis and coagulation necrosis. CDC has a broad spectrum of immunophenotypes. Immunohistochemical analyses revealed CK7, CK19 and CK18 expression in our case, and have also been reported in most of the collecting duct carcinomas. Pheochromocytoma was excluded by: negative immunohistochemistry for Syn and CgA, negativity for OCT3/4 and no lack of INI-1. This made the diagnosis of renal medullary carcinoma unlikely. Positivity for CK7, CK19, and CK18, allowed us to exclude the diagnosis of renal cell carcinoma. Furthermore, the positivity reaction for PAX8, excluded the poorly differentiated urothelial cell carcinoma. Therefore, histopathological examination of the tissue remains as the only precise diagnostic tool.

Clinically, initial diagnosis and treatment decisions for kidney cancer, are often made without histopathological information, based primarily on imaging, and surgical treatment [12]. According to the literature, most CDC cases are high-grade and advanced, but there is currently no consensus on treatment options. Surgery remains the most effective treatment for kidney cancer patients, even those with advanced disease [13]. Surgical procedures include radical nephrectomy and partial nephrectomy [14], but given the high invasiveness of CDC, radical nephrectomy is recommended and may be the only curable opportunity for CDC patients [15, 16]. The literature reports that the average survival of CDC patients after radical nephrectomy is about 6–13 months [17–19].

Most CDCs reported in the literature are less than satisfactory for adjuvant therapy. Orsola [20] et al. reported two CDC cases where adjuvant chemotherapy (doxorubicin + gemcitabine) was administered after radical nephrectomy; however, the mean postoperative survival was only 5.6 months. In Husillos’s study [21], three CDC patients received adjuvant systemic therapy, two received immunotherapy (Sunitinib and Tisimus), and three received a conventional cisplatin chemotherapy regimen. The results showed that the response was very poor, and the survival time was 4 to 7 months However, even with the combination of chemotherapy and targeted therapy, there was no significant improvement in survival time [22]. All these results indicate that adjuvant therapy is not satisfactory for improving the survival rate of patients, the only treatment that seems likely to cure is surgery.

To sum up, collecting duct carcinoma (CDC) is a highly malignant and rare kidney tumor, the biological behavior and the morphological and functional manifestations of which, have unique characteristics. Most patients with CDC have distant metastasis at the time of the initial diagnosis. The prognosis of CDC is generally poor. Moreover, extensive metastasis to the surrounding lymph nodes and refusal of surgical intervention, were considered as mortality predictors.

## Data Availability

The data used to support the findings of this study are included within the article.

## Abbreviations

CDC: Collecting duct carcinoma
